# Investigation of the relationship between pain, fear of movement and falling in geriatric patients

**DOI:** 10.7717/peerj.20661

**Published:** 2026-02-09

**Authors:** Ümmühan Meltem Öztürk, Mümin Polat

**Affiliations:** 1 Health Science Institute/Department of Health and Biomedical Sciences, Burdur Mehmet Akif Ersoy University, Burdur, Turkey; 2 Health Science Faculty/Emergency Aid and Disaster Management Department, Burdur Mehmet Akif Ersoy University, Burdur, Turkey

**Keywords:** Pain, Fear of movement, Fear of falling, Age

## Abstract

**Background:**

Falls among older adults represent a major public health concern and are strongly associated with pain, fear of falling, and fear of movement. Pain may increase fall risk in a dose-response manner, while fear of falling can limit mobility, further enhancing vulnerability. This study aimed to investigate the interrelationship between pain, kinesiophobia, and fear of falling in geriatric patients.

**Methods:**

A descriptive cross-sectional study was conducted in the Physical Therapy Unit of Burdur State Hospital, Turkey, between March 2022 and March 2023. A total of 100 participants aged ≥65 years were recruited by random sampling. Data collection included sociodemographic characteristics, fall history, chronic diseases, and regular medication use. Pain was assessed using the Visual Analog Scale (VAS) and Verbal Category Scale, kinesiophobia using the Tampa Kinesiophobia Scale, and fear of falling using the Tinetti Falls Efficacy Scale. Data were analyzed using Statistical Package for the Social Sciences (SPSS version 23). Descriptive statistics, Student’s *t*-test, analysis of variance (ANOVA) with Tukey *post-hoc*, and Pearson correlation analyses were performed.

**Results:**

The mean age of participants was 70.6 ± 4.5 years. The difference in VAS scores between genders was statistically significant (*p* < 0.05), with higher pain levels in women. A strong positive correlation was found between the Tampa and Tinetti scores (*r* = 0.704, *p* < 0.01), and a moderate positive correlation was observed between VAS and Verbal Category Scale scores (*r* = 0.535, *p* < 0.01). Other subgroup comparisons by education, marital status, and chronic disease were not statistically significant.

**Conclusions:**

Pain, kinesiophobia, and fear of falling are interrelated in older adults and negatively affect daily functioning. Routine assessment of these factors is essential for personalized fall-prevention strategies. Interventions that encourage safe mobility and reduce fear of movement may improve quality of life in the geriatric population.

## Introduction

Aging encompasses progressive, irreversible structural and functional changes that occur at the system, organ, tissue, and cellular levels. The World Health Organization (WHO) defines it as a decreased ability to adapt to environmental stressors, with old age beginning at 65 years of age (Tümerdem, 2006). Increasing life expectancy worldwide has made active aging a global health priority, emphasizing independence in daily activities, preservation of physical function, and prevention of age-related complications (Tambağ, 2013; Howcroft et al., 2017).

Pain, fear of falling, and fear of movement (kinesiophobia) are interrelated problems frequently observed in the geriatric population. Pain has been shown to have a dose–response relationship with falls, whereby higher pain intensity is associated with increased fall risk (Stubbs et al., 2014; Gell & Patel, 2019). Fear of falling, even in the absence of previous falls, is prevalent among older adults and can lead to activity restriction, functional decline, and increased frailty (Legters, 2002; Zijlstra et al., 2007; Scheffer et al., 2008). Similarly, kinesiophobia may result in avoidance of movement due to fear of pain or re-injury, which in turn perpetuates inactivity, muscle weakness, and reduced balance (Tunca Yılmaz et al., 2011; Vincent et al., 2013; Merchant et al., 2022). These behavioral responses contribute to a vicious cycle that exacerbates fall risk and diminishes quality of life in older adults.

Recent peer-reviewed evidence further emphasizes the multidimensional nature of pain-related movement fear. A comprehensive scoping review published in *PeerJ* identified key conceptual and methodological trends regarding kinesiophobia among health professionals, highlighting its clinical relevance across diverse practice settings (Doutre et al., 2024). Consistently, Ertem (2025) demonstrated that individuals with ankylosing spondylitis exhibit significantly higher levels of kinesiophobia, which are closely associated with greater pain intensity and functional impairment. Moreover, Chantanachai et al. (2022) examined fall risk factors among community-dwelling older adults with mild cognitive impairment, reporting that both psychological and physical domains jointly contribute to fall susceptibility. Together, these recent studies reinforce the interrelationship between pain, fear of movement, and fall risk in geriatric populations.

Given the complexity of fall risk, recent research highlights the need for personalized preventive approaches that address not only physical but also psychological and behavioral factors (Delbaere et al., 2004; Todd & Skelton, 2004). Despite the recognition of these associations, the combined relationship between pain, fear of falling, and fear of movement in older adults remains underexplored, particularly in community-dwelling populations in Turkey.

Therefore, the present study aimed to investigate the interrelationships between pain, kinesiophobia, and fear of falling in individuals aged 65 years and older. By identifying how these factors interact, we sought to contribute to the development of targeted strategies for fall prevention and quality-of-life improvement in the geriatric population.

## Materials & Methods

### Study design and setting

This study was designed as a descriptive cross-sectional study and was conducted between March 2022 and March 2023 in the Physical Therapy Unit of Burdur State Hospital, located in southwestern Turkey. The study was to evaluate the interrelationship between pain, kinesiophobia, and fear of falling in geriatric patients.

### Participant sampling

A total of 213 patients aged 65 years and above who applied to the physical therapy outpatient clinic were evaluated for eligibility. Participants were selected using a simple random sampling method. The required sample size was estimated using G*Power version 3.1, with an effect size of 0.40, *α* = 0.05, and power (1–*β*) = 0.90. The calculation was based on an analysis of variance (ANOVA) fixed effects model. The estimated sample size was 102, considering 20% attrition; the final sample included 100 participants after exclusions due to incomplete data.

### Inclusion and exclusion criteria

Participants were included in the study if they were aged 65 years or older, were able to walk independently or with the use of assistive devices, had no diagnosed cognitive impairment or neurological disorders such as Alzheimer’s disease or Parkinson’s disease, and voluntarily agreed to participate. Individuals were excluded from the study if they had an acute illness during the data collection period, had severe visual or hearing impairments that could hinder questionnaire completion, or refused to provide informed consent.

### Participant demographics

The sample consisted of 100 individuals (mean age: 70.58 ± 4.54 years). Sociodemographic data collected included gender, age, marital status, education level, occupation, dominant body side, history of surgery, presence of chronic diseases, and regular use of medication. Body mass index (BMI) was calculated using reported height and weight.

### Data collection procedure

Data were collected through face-to-face structured interviews conducted by the lead researcher in a private consultation room within the hospital. The survey tools were administered in the same order for all participants to ensure consistency. Each session lasted approximately 20–30 min. Participants were encouraged to ask questions if they did not understand any items.

### Measurement instruments

Fear of falling was assessed using the Tinetti Falls Efficacy Scale (FES), a validated instrument widely used in older populations. Fear of movement was evaluated with the Tampa Scale for Kinesiophobia (TAMPA), for which the Turkish validity and reliability have previously been established. Pain intensity was measured using two standardized tools: the Visual Analog Scale (VAS) and the Verbal Category Scale (VCS), both of which are commonly used in geriatric populations. In addition, a sociodemographic data form was used to collect information on age, gender, marital status, education level, occupation, fall history, comorbidities, and regular medication use.

### Ethical considerations

Ethical approval was obtained from the Burdur Mehmet Akif Ersoy University Clinical Research Ethics Committee (Approval No: YL21-486). Institutional permission was granted by the Burdur State Hospital administration. All participants provided written informed consent prior to inclusion in the study. Participation was voluntary, and individuals could withdraw at any stage without any consequences.

### Statistical analysis

Data were analyzed using Statistical Package for the Social Sciences (SPSS) version 23. Normality was assessed with the Kolmogorov–Smirnov test. Descriptive statistics were calculated as mean ± standard deviation and frequency (%). Group comparisons were performed using Student’s *t*-test and one-way ANOVA with Tukey’s *post-hoc* tests. Bivariate associations among scale scores were examined with Pearson correlation coefficients. A two-sided *p* < 0.05 was considered statistically significant. Multivariable regression was not conducted in the present study.

## Results

Detailed descriptive statistics are presented in Tables 1–3; below we summarize only the key findings. A total of 100 individuals participated in the study. The mean age was 70.58 ± 4.54 years. The mean height was 165.09 ± 0.6 cm, and the mean weight was 77.62 ± 7.03 kg. The average symptom duration was 3.79 ± 1.09 years (Table 1).

**Table 1:** Sociodemographic and clinical characteristics of participants.

Independent variable	The dependent variable	*n*	%
Gender	Woman	54	54
	Male	46	46
Dominant Party	Right	89	89
	Left	11	11
Marital status	Married	80	80
	Single	20	20
Education status	Primary school	38	38
	Middle school	23	23
	High school	39	39
Job	Housewife	27	27
	Retired	73	73
Surgical Presence	There is	28	28
	None	72	72
Chronic Disease	There is	75	75
	None	25	25
Continuous Drug Use	There is	76	76
	None	24	24
Affected Area	Right	14	14
	Left	8	8
	bilateral	78	78

**Notes.**

Weight (kg), height (cm), and Body Mass Index (BMI, kg/m^2^) values have been added to Table 1 to provide a complete description of participants’ anthropometric characteristics. BMI was calculated as weight (kg) divided by height squared (m^2^).

Group comparisons showed that female participants reported higher VAS pain scores than males (*p* < 0.05), whereas Tinetti, TAMPA/TSK, and VCS scores did not differ significantly by sex (*p* > 0.05). No significant differences were observed across dominant side, marital status, education, occupation, chronic disease, or medication use. Participants with a history of surgery had higher mean values across scales, but these did not reach statistical significance. See Table 2 for full estimates. These subgroup comparisons (Table 2) align with the study’s main objective by illustrating how demographic and clinical factors may influence the interplay between pain, fear of movement, and fear of falling in older adults.

**Table 2:** Distribution of scale scores by participant characteristics.

Variable	N	%
Left knee	5	5
Right knee	7	7
Bilateral knee	17	17
Waist area	21	21
Neck area	11	11
Neck and shoulder area	11	11
Right shoulder area	5	5
Left shoulder area	5	5
Both hands	5	5
Waist and left leg area	7	7
Waist and right leg area	6	6

**Table 3:** Subgroup comparisons of scale scores according to demographic and clinical variables.

Independent variable		The dependent variable	*n*	Mean	Std. Giant.	t/f	*P*
Gender	Woman	Tampa	54	45.65	5.11	−1.26	0.21
	Male		46	46.89	4.72		
	Woman	Tinetti	54	49.19	6.50	0.03	0.98
	Male		46	49.15	5.39		
	Woman	Vas	54	5.13	0.85	2.32	0.02
	Male		46	4.74	0.83		
	Woman	Pain Assessment	54	5.61	1.35	1.46	0.15
	Male		46	5.24	1.18		
Dominant Party	Right	Tampa	89	46.15	4.62	−0.42	0.67
	Left		11	46.82	7.35		
	Right	Tinetti	89	49.09	5.83	−0.38	0.71
	Left		11	49.82	7.44		
	Right	Vas	89	4.98	0.87	0.91	0.36
	Left		11	4.73	0.79		
	Right	Pain Assessment	89	5.46	1.32	0.46	0.65
	Left		11	5.27	0.90		
Marital status	Married	Tampa	80	46.23	4.94	0.02	0.98
	Single		20	46.20	5.11		
	Married	Tinetti	80	49.29	5.63	0.39	0.70
	Single		20	48.70	7.38		
	Married	Vas	80	4.95	0.86	0.00	1.00
	Single		20	4.95	0.89		
	Married	Pain Assessment	80	5.49	1.28	0.74	0.46
	Single		20	5.25	1.29		
Education status	Primary school	Tampa	38	46.26	5.53	0.03	0.97
	Middle school		23	46.39	5.37		
	High school		39	46.08	4.14		
	Primary school	Tinetti	38	49.92	6.61	0.48	0.62
	Middle school		23	48.70	5.63		
	High school		39	48.72	5.61		
	Primary school	Vas	38	5.13	0.88	1.39	0.25
	Middle school		23	4.83	0.78		
	High school		39	4.85	0.87		
	Primary school	Pain Assessment	38	5.50	1.27	0.22	0.80
	Middle school		23	5.52	1.47		
	High school		39	5.33	1.20		
Job	Housewife	Tampa	27	44.85	5.07	−1.70	0.09
	Retired		73	46.73	4.84		
	Housewife	Tinetti	27	49.22	6.97	0.05	0.96
	Retired		73	49.15	5.63		
	Housewife	Vas	27	5.19	0.79	1.68	0.10
	Retired		73	4.86	0.87		
	Housewife	Pain Assessment	27	5.33	1.11	−0.50	0.62
	Retired		73	5.48	1.34		
Surgical Presence	There is	Tampa	28	46.75	4.80	0.67	0.51
	None		72	46.01	5.02		
	There is	Tinetti	28	49.36	6.44	0.19	0.85
	None		72	49.10	5.85		
	There is	Vas	28	5.14	0.89	1.41	0.16
	None		72	4.88	0.84		
	There is	Pain Assessment	28	5.79	1.32	1.70	0.09
	None		72	5.31	1.25		
Chronic Disease	There is	Tampa	75	46.47	4.98	0.86	0.39
	None		25	45.48	4.85		
	There is	Tinetti	75	49.13	6.05	−0.11	0.92
	None		25	49.28	5.89		
	There is	Vas	75	4.97	0.84	0.47	0.64
	None		25	4.88	0.93		
	There is	Pain Assessment	75	5.49	1.23	0.72	0.47
	None		25	5.28	1.43		
Continuous medicine	There is	Tampa	76	46.42	4.97	0.72	0.47
	None		24	45.58	4.92		
	There is	Tinetti	76	49.09	6.02	−0.23	0.82
	None		24	49.42	5.98		
	There is	Vas	76	4.97	0.83	0.49	0.63
	None		24	4.88	0.95		
	There is	Pain Assessment	76	5.53	1.26	1.20	0.23
	None		24	5.17	1.34		
Affected Area	Right	Tampa	14	48.07	4.65	1.207	0.3
	Left		8	46.5	6		
	bilateral		78	45.86	4.87		
	Right	Tinetti	14	48.93	5.14	0.124	0.88
	Left		8	48.25	5.78		
	bilateral		78	49.30	6.20		
	Right	Vas	14	4.85	1.03	0.101	0.9
	Left		8	5	0.93		
	bilateral		78	4.96	0.83		
	Right	Pain Assessment	14	5.35	1.22	0.319	0.73
	Left		8	5.12	1.13		
	bilateral		78	5.48	1.32		

The distribution of affected regions is shown in Table 3. The distribution of affected regions was as follows: left knee, right knee, both knees, lower back, neck, neck and shoulder, right shoulder, left shoulder, both hands, lower back and left leg, and lower back and right leg (Table 3).

When the gender variable was evaluated, the mean TAMPA score was higher among male participants, while the mean scores of the Tinetti, VAS, and Pain Assessment scales were higher among female participants. While the differences in TAMPA, Tinetti, and Pain Assessment scores were statistically insignificant (*p* >  0.05), the difference in VAS scores was statistically significant (*p* < 0.05).

When the dominant side variable was assessed, the mean VAS and Pain Assessment scores were higher on the right side, whereas TAMPA and Tinetti scores were higher on the left side. The differences in the scale scores based on dominant side were statistically insignificant (*p* > 0.05).

For the marital status variable, the mean VAS scores were equal in both married and single participants. TAMPA, Tinetti, and Pain Assessment scores were higher on average among married participants. The differences between these scores and marital status were not statistically significant (*p* > 0.05).

Regarding educational attainment, the mean VAS and Tinetti scores were higher among those with primary school education, while TAMPA and Pain Assessment scores were higher among those with secondary school education. These differences were statistically insignificant (*p* > 0.05).

In the comparison of occupational status with the dependent variables, TAMPA and Pain Assessment scores were higher among retired participants, while Tinetti and VAS scores were higher among housewives.

For the surgical history variable, those who had undergone surgery had higher mean scores in TAMPA, Tinetti, VAS, and Pain Assessment compared to those who had not.

When chronic disease was evaluated, participants with chronic illness had higher TAMPA, VAS, and Pain Assessment scores, while participants without chronic disease had higher Tinetti scores. These differences were not statistically significant (*p* >  0.05).

In terms of regular medication use, participants who continuously used medication had higher TAMPA, VAS, and Pain Assessment scores than those who did not. The Tinetti score was higher among those who did not use medication regularly. These differences were statistically insignificant (*p* > 0.05).

Regarding the affected region, higher TAMPA scores were observed in those with right-side involvement, higher VAS scores in those with left-side involvement, and higher Tinetti and Pain Assessment scores in those with bilateral involvement. These differences were also not statistically significant (*p* > 0.05).

### Scale correlations (Pearson correlation)

A positive correlation was found between TAMPA and Tinetti scores (+0.704). The correlation between TAMPA and VAS scores was negative (−0.176), as was the correlation between TAMPA and Pain Assessment scores (−0.129). The correlation between Tinetti and VAS scores was also negative (−0.224), as was the correlation between Tinetti and Pain Assessment scores (−0.221). A positive correlation was found between VAS and Pain Assessment scores (+0.535) (Table 4). Pearson correlations between scales are provided in Table 4, which summarizes the strength and direction of associations among pain, fear of movement, and fear of falling measures. Significant relationships are highlighted in the text where relevant.

**Table 4:** Pearson correlation coefficients between scale scores.

**Variable 1**	**Variable 2**	**r**	***p***
TAMPA	Tinetti	0.704	<0.01
TAMPA	VAS	−0.176	0.08
TAMPA	VCS	−0.129	0.12
Tinetti	VAS	−0.224	0.06
Tinetti	VCS	−0.221	0.07
VAS	VCS	0.535	<0.01

**Notes.**

Pearson correlation coefficients (r) and corresponding *p*-values are presented.

TAMPA Tampa Scale for Kinesiophobia FES Falls Efficacy Scale VAS Visual Analog Scale VCS Verbal Category Scale

Subgroup analyses were conducted for gender, dominant side, marital status, education level, and occupation because these variables are well-documented risk factors for falls in older adults (Todd & Skelton, 2004; Masud & Morris, 2001). However, no significant differences were observed across subgroups (*p* > 0.05).

## Discussion

This study examined the interrelationship between pain, fear of movement, and fear of falling in older adults. Our results indicated that female participants reported significantly higher pain scores compared to males, while no significant differences were observed in Tinetti, TAMPA, or pain assessment scales. These findings are consistent with previous reports that women are more likely to experience chronic pain and falls compared to men (Todd & Skelton, 2004). The relationship between gender and fall risk highlights the need for gender-sensitive prevention strategies.

Pain is a well-established determinant of falls in the elderly. Consistent with previous studies, our results support the notion that pain contributes to reduced activity, balance problems, and higher fall risk (Stubbs et al., 2014; Gell & Patel, 2019). The observed positive correlation between pain severity and fear-related outcomes suggests that pain not only impairs mobility but also exacerbates psychological concerns, reinforcing the cycle of inactivity and vulnerability. Fear of movement (kinesiophobia) and fear of falling are maladaptive responses commonly observed among older adults. In our study, TAMPA scores showed a strong correlation with Tinetti scores, confirming earlier research indicating that fear of movement limits participation in daily activities and increases fall risk (Zijlstra et al., 2007; Scheffer et al., 2008; Tunca Yılmaz et al., 2011). Moreover, avoidance behaviors associated with fear of falling have been shown to contribute to further functional decline, reduced mobility, and frailty. Our findings emphasize that interventions should not only target physical risk factors but also address psychological dimensions. The subgroup analyses presented in Table 2 further support the main objective of our study by demonstrating that sociodemographic and clinical variables, including gender, education level, and chronic disease status, may influence pain and movement-related fear. Although these associations were not statistically significant, the observed tendencies align with the conceptual model linking physical and psychological domains in fall risk among older adults. Interestingly, although both VAS and VCS are validated pain assessment tools, only VAS scores demonstrated statistical significance. This discrepancy may be attributed to the continuous nature and higher sensitivity of the VAS, which allows for finer gradations of pain intensity compared to the categorical VCS. Additionally, older adults may perceive and report pain differently when constrained to verbal categories, potentially leading to less variability in VCS scores. The strong positive correlation between TAMPA and Tinetti scores highlights the reciprocal relationship between movement fear and functional performance. This suggests that interventions aiming to reduce kinesiophobia could also enhance balance confidence and mobility, which are critical components in fall prevention programs.

Although subgroup analyses for chronic disease did not reveal statistically significant differences, trends were in line with previous findings. Chronic illness has been associated with greater fatigue, reduced activity, and impaired balance (Karataş & Maral, 2010; Schnelle et al., 2012). Fatigue in elderly populations has also been linked to sleep disturbances and lack of physical exercise (Schultz-Larsen & Avlund, 2007). Interventional studies have shown that structured physical activity improves sleep quality and reduces fatigue (Ferris et al., 2005; Reid et al., 2010). Considering that fatigue and poor sleep are also linked to fall risk and mortality (Bloom, Ahmed & Alessi, 2009; Onat et al., 2013), these parameters warrant greater attention in geriatric assessments.

Physical activity plays a critical role in maintaining balance and mobility. Previous studies demonstrated significant improvements in strength and balance following structured exercise interventions (Lee & Park, 2013; Soyuer, Şenol & Şenol, 2012). In our study, although occupation and activity levels were not significant predictors, trends supported the established literature that sedentary individuals have poorer outcomes compared to physically active peers (Fortuño-Godes, Guerra-Balic & Cabedo-Sanromà, 2013). Taken together, these findings suggest that exercise-based interventions should be prioritized for fall prevention.

The use of assistive devices is considered both a risk factor and a preventive strategy. While elderly individuals with gait or balance impairments are more prone to falls, assistive devices can help stabilize mobility when appropriately prescribed (Fink, Wyman & Hanlon, 2003; Rao, 2005; Erdoğmuş & Tüzün, 2001). Our findings support the view that careful assessment and tailored prescription of assistive devices are critical for reducing fall risk.

Falls remain a major public health concern. Globally, they are among the leading causes of mortality and morbidity in older adults (Berg et al., 1997; Tideiksaar, 1988). Fear of falling has been reported in 12–65% of community-dwelling elderly without prior falls, and up to 92% among those with a history of falls (Legters, 2002). This highlights the substantial psychological burden even beyond actual fall events. Our findings align with the broader literature, showing that pain, psychological factors, and physical inactivity together perpetuate fall risk in aging populations.

This study has some limitations, including its cross-sectional design, single-center setting, and relatively small sample size, which may reduce generalizability and statistical power. Nevertheless, the integration of multiple scales (VAS, VCS, TAMPA, Tinetti) provided a comprehensive evaluation of pain, fear of movement, and fear of falling. Future longitudinal and interventional studies are needed to establish causal relationships and evaluate the effectiveness of multidisciplinary programs.

In conclusion, this study demonstrated that pain intensity, fear of movement, and fear of falling are interrelated constructs that contribute to mobility limitations in older adults. Although some associations did not reach statistical significance, the observed trends suggest that increased pain and kinesiophobia may negatively affect functional performance and confidence in balance. These findings emphasize the importance of comprehensive geriatric assessments that address both physical and psychological factors in fall prevention. Future research with larger and more diverse samples, including regression-based analyses, is recommended to clarify the causal pathways between pain, movement fear, and fall risk.

## Conclusions

Aging is one of the health-related challenges that affects all body systems particularly the musculoskeletal system resulting in a sense of inadequacy, disability, and loss of workforce, and is becoming increasingly prevalent. While technological advancements continue to make daily life easier for individuals, they also contribute to a sedentary lifestyle and reduced levels of physical activity. Furthermore, risk factors such as smoking, obesity, poor posture, and strenuous working conditions are known contributors to chronic pain. Over time, impaired posture, muscle weakness, spasms, and pain diminish the quality of life and the ability to perform daily activities among geriatric patients. Cognitive functions and emotional states are also significantly affected in patients experiencing chronic pain, leading to delays in the initiation of rehabilitation.

Among the preventive measures for chronic conditions, proper nutrition programs stand out as essential. A balanced and healthy diet increases resistance to disease and significantly lowers the risk of illness.

Fear of movement, also known as kinesiophobia, has recently drawn attention from researchers as a factor contributing to persistent pain and the limitation of daily activities in geriatric patients. Pain in elderly individuals often cannot be explained by clinical findings alone. Instead, behavioral, social, perceptual, and physical factors are believed to play a role. Reduced physical activity leads to decreased physical fitness, which in turn may contribute to the development of diabetes, obesity, and cardiovascular diseases. Additionally, living in social isolation often leads to psychological problems in elderly individuals.

Identifying kinesiophobia in geriatric patients and understanding its connection with quality of life are critical steps toward improving their well-being and promoting activity participation. Encouraging appropriate physical activity among elderly individuals can help reduce sedentary behavior and mitigate the associated health risks. It is well established that elderly individuals who exercise regularly experience lower levels of kinesiophobia. Therefore, engaging elderly individuals in regular group-based exercise programs can increase participation and adherence. Educational initiatives focused on specific health topics can also raise awareness and positively influence the quality of life. Elderly individuals who become more informed tend to incorporate this knowledge into their daily lives, improving self-care.

Additionally, those who engage in their own housework remain more physically active throughout the day. Thus, encouraging older adults to perform household tasks is important for maintaining daily activity levels.

This study demonstrated that pain intensity and kinesiophobia are important correlates of fear of falling among older adults. Although fear of movement and falling are common, they are modifiable through appropriate interventions. Incorporating regular exercise, balance training, and psychological support into geriatric care may reduce these fears and improve quality of life. Future studies using longitudinal and intervention designs could further clarify these relationships. These findings collectively emphasize the multifactorial nature of pain, movement fear, and fall risk in older adults. Addressing these interrelated factors through targeted physical activity programs and psychosocial support could help improve mobility and reduce fall incidence in geriatric populations.

### Limitations

This study has several limitations. First, the cross-sectional design prevents causal inferences. Second, the single-center setting may limit the generalizability of findings. Third, although bivariate analyses provided meaningful insights, regression models were not performed to adjust for potential confounders such as age, gender, and comorbidities. Future research could incorporate regression-based models to better adjust for potential confounding variables. Finally, the sample size was relatively modest, which may have limited the detection of smaller effects. Future studies should employ multivariable regression models to adjust for potential confounding factors (*e.g.*, age, sex, comorbidities) and to test the robustness of the observed associations.

### Future research

Future research should further investigate the causal pathways between pain, kinesiophobia, and fear of falling in older adults. Longitudinal and interventional studies are needed to determine whether reducing pain and movement fear can directly lower fall risk. Moreover, integrating objective mobility measures and psychosocial interventions into future designs would help clarify how physical and psychological factors interact in fall prevention.

##  Supplemental Information

10.7717/peerj.20661/supp-1 Supplemental Information 1 Dataset and codebook

10.7717/peerj.20661/supp-2 Supplemental Information 2 STROBE checklist

## Additional Information and Declarations

### Data Availability

The following information was supplied regarding data availability: The raw data is available in the Supplementary File.
